# Detection of live *M. bovis* BCG in tissues and IFN-γ responses in European badgers (*Meles meles*) vaccinated by oropharyngeal instillation or directly in the ileum

**DOI:** 10.1186/s12917-019-2166-4

**Published:** 2019-12-06

**Authors:** Sandrine Lesellier, Maria-Laura Boschiroli, Jacques Barrat, Christoph Wanke, Francisco J. Salguero, Waldo L. Garcia-Jimenez, Alex Nunez, Ana Godinho, John Spiropoulos, Simonette Palmer, Dipesh Dave, Paul Anderson, Jean-Marc Boucher, Krystel de Cruz, Sylvie Henault, Lorraine Michelet, Sonya Gowtage, Gareth A. Williams, Allan K. Nadian, Elodie Monchâtre-Leroy, Frank Boué, Mark A. Chambers, Céline Richomme

**Affiliations:** 1 Animal and Plant Health Agency, New Haw, UK; 2 Laboratory for Animal Health, Tuberculosis National Reference Laboratory, University Paris-Est, Anses, Maisons-Alfort, France; 3 Anses, Nancy laboratory for rabies and wildlife, Malzéville, France; 4 Medimetrics Personalized Drug Delivery B.V., High Tech Campus 10, 5656 AE Eindhoven, The Netherlands; 5 University of Surrey, Guildford, UK; 6 Public Health England, Porton Down, UK

**Keywords:** BCG, Badger, Vaccine, Mucosa, Capsule, Tonsil, Lymphatic drainage

## Abstract

**Background:**

Oral vaccination with *Mycobacterium bovis* Bacille of Calmette and Guerin (BCG) has provided protection against *M. bovis* to badgers both experimentally and in the field. There is also evidence suggesting that the persistence of live BCG within the host is important for maintaining protection against TB. Here we investigated the capacity of badger inductive mucosal sites to absorb and maintain live BCG. The targeted mucosae were the oropharyngeal cavity (tonsils and sublingual area) and the small intestine (ileum).

**Results:**

We showed that significant quantities of live BCG persisted within badger in tissues of vaccinated badgers for at least 8 weeks following oral vaccination with only very mild pathological features and induced the circulation of IFNγ-producing mononuclear cells. The uptake of live BCG by tonsils and drainage to retro-pharyngeal lymph nodes was repeatable in the animal group vaccinated by oropharyngeal instillation whereas those vaccinated directly in the ileum displayed a lower frequency of BCG detection in the enteric wall or draining mesenteric lymph nodes. No faecal excretion of live BCG was observed, including when BCG was delivered directly in the ileum.

**Conclusions:**

The apparent local loss of BCG viability suggests an unfavorable gastro-enteric environment for BCG in badgers, which should be taken in consideration when developing an oral vaccine for use in this species.

## Background

Tuberculosis (TB), caused by *Mycobacterium bovis,* is an important infectious disease affecting cattle worldwide [1] and in particular in the UK and Ireland where its eradication is hampered by reservoirs of *M. bovis* in sympatric European badger (*Meles meles*) [2–4]. The transmission of TB from badgers cannot be excluded in other part of Europe such as Spain [5] or France [6]. Vaccinating badgers against TB has the potential for diminishing the risk of *M. bovis* transmission between badgers and cattle by reducing the level of *M. bovis* infection and excretion in badger populations. In addition, vaccination does not increase epidemiological risks for herds associated with social group structure disrupture caused by culling, because of enhanced activities by infected badgers in the proximity of cattle pastures and buildings [7]. *M. bovis* Bacillus Calmette and Guérin (BCG) is the only vaccine currently licensed for use in humans against TB and one of the most widely used vaccines in the world [8]. BCG was licensed for intramuscular vaccination of badgers in the UK in 2010 (BadgerBCG, AJ vaccines, Denmark). However, a vaccine that could be orally administered in bait would be better suited for use in wildlife as it would be easier to deploy than an injectable vaccine [9]. The ability of oral BCG to confer protection was demonstrated in humans, including new-borns, by Albert Calmette and Camille Guérin in the 1920s [10]. Although the intra-dermal (ID) route became more frequently used subsequently [8, 11], oral vaccination continued in Brazil with the Moreau BCG strain [12, 13]. There is now a renewed interest in mucosal vaccination against TB (oral and intra-nasal) as it can be a more efficient trigger of protective pulmonary responses against TB [14–20].

Badgers vaccinated orally with BCG were protected against the development of TB lesions, compared with non-vaccinated controls, in experimental studies [21–23] and in the field [24]. Palatable baits have been developed for the oral delivery of BCG to badgers [25, 26]. However, the degree of protection conferred by oral BCG generally appears to be more variable than when BCG is delivered parenterally as described in [23, 27]. Consequently, further research and developmental work are required to produce a more consistently protective oral BCG vaccine.

Efficient mucosal uptake of oral vaccines followed by antigen presentation are considered crucial for protection [28] and the persistence of live BCG in the host following vaccination appears to be a requirement for a long-term protective effect, at least in mice [29–31]. The capacity of badger mucosal tissues to absorb and maintain live BCG is unknown. To study this, we targeted two main inductive digestive mucosal sites: the oropharyngeal cavity, including the tonsils; and the ileum. Live BCG was delivered in the ileum after bypassing the stomach using an electronic drug delivery capsule: IntelliCapFR® (Fast Release) (Medimetrics, The Netherlands) [32]. Here, we report its first use to deliver a live vaccine under controlled release in the enteric lumen of a group of captive animals. Lipids may contribute to mucosal uptake and protective immunogenicity of BCG in badgers [21–23] and in other species such as mice [33–35], guinea pigs [36], cattle [37], and deer [38] and could form part of an efficacious vaccine formulation for badgers. Lipids were added to BCG in study 2. The two studies were conducted to assess if the BCG delivery should target the gut more efficiently than with the current bait. This bait is masticated and BCG is absorbed by the oral mucosa or by the intestine following gastric transit. BCG doses and formulations in both studies were compatible with the size and composition of the lead bait candidate [26].

## Methods

### Animals

A total of twenty adult badgers were trapped from a North-Eastern area of France, with no recent TB outbreaks [39], on two separate occasions. France is designated as officially free from bovine TB by the European Commission Decision 2003/467/EC. Two studies were conducted comprising groups A and B (*N* = 6, each) for study 1, and groups C and D (*N* = 4, each) for study 2 (Table 1) with a random allocation of the pens (containing two to four animals each) to each treatment. Study 1 followed study 2 with refinement in the protocols in study 1 to increase the scientific output (two more samples were collected: faeces and tracheal wash, and histology and ELISPOT were conducted). The animals were not randomly distributed between pens; badgers cannot be easily mixed as they are attached to their original social group and animals remained in their original group. The small number of animals was adapted to the accommodation size, and considered sufficient for providing exploratory data. The badgers were housed in four covered outdoor pens, each containing a wooden sett and environmental enrichment; animals of different treatments were not mixed within pen. The studies were ethically authorised by the Agence Nationale de sécurité sanitaire de l’alimentation, de l’environnement et du travail (Anses), Ecole Nationale Vétérinaire d’Alfort (ENVA), University Paris-Est Créteil (UPEC), Anses/ENVA/UPEC Ethical Committees on behalf of the Ministry of Research (Avis n°11/11/15–5 et 13/12/11–11, n° de dossier 11–0065 et 11–0065 bis) under Anses agreement C 54–431-1, and by the Animal and Plant Health Agency (APHA) Ethics Committee in the UK. ARRIVE guidelines for reporting animal research [40] have been followed as much as possible. The welfare of the animals was monitored remotely using infra-red (IR) cameras and by direct observation. All animals were identified individually using electronic microchips (Trovan®, UK) inserted subcutaneously between the shoulder blades before the start of the study. The badgers remained in good clinical condition throughout the study apart from one group A badger (diagnosed with cutaneous *Histoplasma capsulatum* (histoplasmosis) with lesions detected prior to the start of the study) and one group D badger (multicentric T cell lymphoma, stage IV, diagnosed at post-mortem in the hepatic and mesenteric lymph nodes). One group A badger had pulmonary infection with *Emmonsia crescens* (adiaspiromycosis) diagnosed at post-mortem. Captivity and vaccination did not compromise pregnancy in four females (one animal in each group).

**Table 1:** Summary of vaccination protocols and tests (bacteriology, histology and immunology)

		Groups			
		Group A	Group B	Group C	Group D
Number of animals		6	6	4	4
Study		1		2	
Vaccine delivery		Intellicap capsule	Oropharyngeal	Oropharyngeal	Oropharyngeal
Lipid added		No		HPO	Cocoa Butter
Mucosal target		Intestine	Oral	Oral	Oral
Volume of vaccine	Ileum	300 μl			
	Tonsils		100 μl	200 μl	200 μl
	Sub-lingual		100 μl		
Vaccine dose (CFU)		5.67 × 10^7^		6.73 × 10^7^	
Culture sample		Section	Section	Complete	Complete
Inoculum volume per 7H11 agar plate		400 μl		100 μl	
Volume of saline added per sample		3, 5 or 10 ml saline to 0–0.5 g, 0.5–1 g and > 1 g tissues, respectively		10 ml saline	
RT-PCR primers		IS*6110*, IS*1081*, IS*1561*’, *hsp65, RD1* flanking region		IS*6110*, IS*1081*, IS*1561*’, RV1510	
Tissues pooled		None		Left and right tonsils (pool 1), retro-pharyngeal (pool 2) and mandibular (pool 3) lymph nodes	
Histology		Yes		No	
Tracheal section wash		Yes		No	
Environmental faecal sample		Yes		No	
IGRA		Yes		Yes	
ELISPOT		Yes		No	

The animals were housed for up to 28 weeks before vaccination. For both vaccination and euthanasia, each badger was immobilised with ketamine hydrochloride (~ 10 mg/kg) (Imalgene 1000®, Merial, Lyon, France) and medetomidine hydrochloride (~ 0.1 mg/kg) (Domitor®, Pfizer animals health, Exton, PA, USA), co-administered by intra-muscular injection with a pole syringe (Geniaplex 5 mL with 1 m extension, Genia, Hilaire de Chaléons, France). The anaesthetised animals were transported to a laboratory facility located approximately 10 m from the pens and were clinically examined before the start of any procedure to keep records of the animal welfare. Blood was collected from the jugular vein into 10 ml heparinised vacutainer tubes (BD®, Becton, Dickinson and Company, Franklin Lakes, UK) before any other procedure. Vaccination at the beginning of the study or IV delivery of an overdose (~ 15 ml per badger) of barbiturates (T-61 Euthanasia Solution®, Merk Animal Health, Milton Keynes, UK) for euthanasia 8 weeks post-vaccination were then conducted. Following euthanasia, a detailed post-mortem examination was carried out.

### Vaccine

All the badgers were vaccinated with *M. bovis* BCG, Danish strain 1331 (“Concentrated Bulk BCG”; Statens Serum Institute, now AJ Vaccines, Denmark). BCG was suspended in 88.7 mM sodium 2-aminopentanedioate (monosodium glutamate, MSG) and stored at 4–8 °C until use. The BCG doses at vaccination were determined by incubation on Modified Middlebrook 7H11 solid agar for 4 weeks at 37 °C: they were 5.67 × 10^7^ CFU (SEM: 1.73 × 10^6^) for groups A and B and 6.73 × 10^7^ CFU (SEM: 8.82 × 10^6^) for groups C and D (Table 1) and not statistically different from each other (Mann-Whitney test, *p* > 0.05).

### Vaccine delivery using IntellicapFR® capsules

**Fig. 1:**
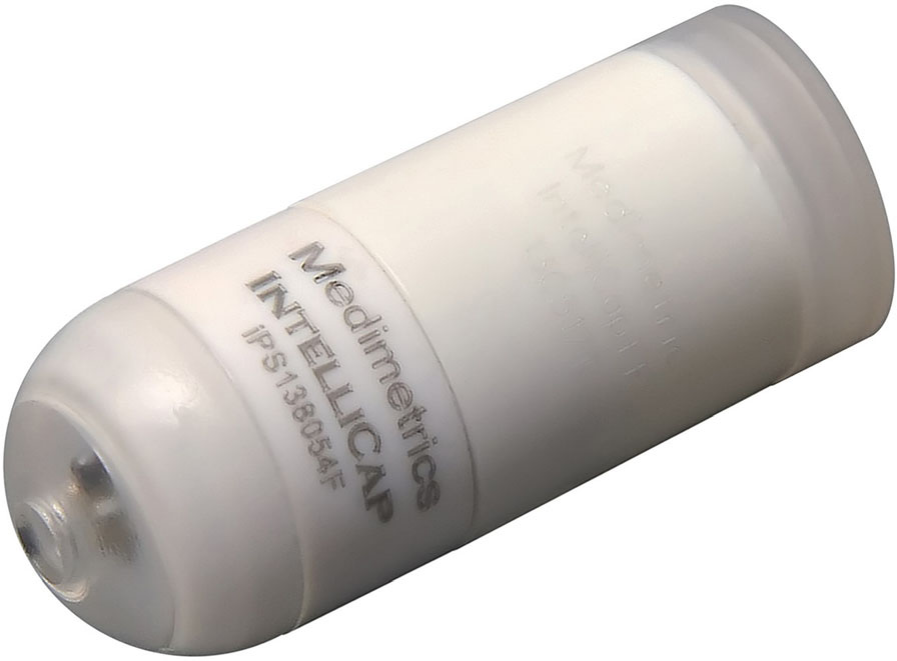
IntellicapFR capsules

The IntellicapFR® capsule (Medimetrics, The Netherlands) is composed of two parts: a container and microelectronic body (Fig. 1). The microfluidic medication container is suitable for liquid or solid volumes ranging from 300 to 700 μl for a total size of approximately 27 × 11 × 11 mm. The microelectronic body of the capsule includes a battery, a real time communication unit, a microcomputer, an actuator for payload release and a monitoring sensor that records pH and temperature. These data are transmitted to a command computer via an intermediate receiver. Upon remote activation at the target region in the gastric-intestinal tract, a gas cell within the capsule generates hydrogen which expels the payload from the reservoir. The pH sensor was calibrated before introduction of the capsule and after capsule excretion and recovery, using commercially available buffers (pH 1, 4, and 7).

The survival of BCG inside the capsules was evaluated in vitro. Six capsules were filled with 300 μl of BCG suspension, immersed individually in 50 ml sterile conical centrifuge tubes containing MSG solution, and agitated for a total of 20 h, either under a ‘strong’ motion using a blood tube rotator (three capsules) or under ‘gentle’ motion using a gyratory rocker (30 rpm) (three capsules). The capsules were then electronically activated to release their BCG payload. The concentration of BCG in the solutions surrounding the capsules before and after payload release was measured by culture.

**Fig. 2:**
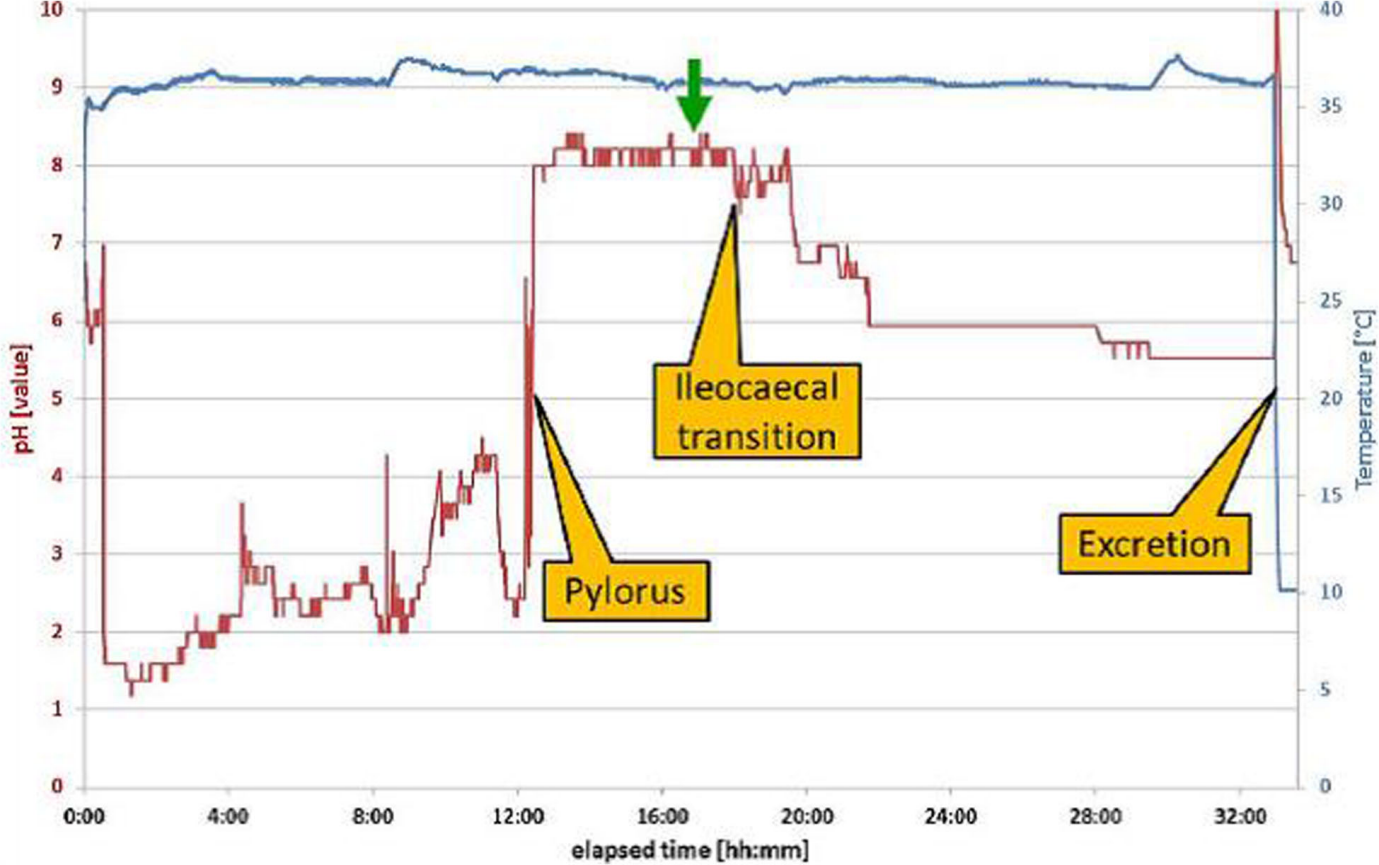
pH and temperature profile for badgers 7D63. The green arrow indicates the time of the capsule activation

Group A badgers (*n* = 6) were vaccinated with BCG released by IntellicapFR® capsules (Table 1), each loaded with 300 μl of BCG suspension. Using a purpose-made delivery system (Medimetrics, the Netherlands), each capsule was individually introduced in the distal oesophagus or the stomach of each of the anaesthetised badgers. The animals were then taken back to their pens to recover from general anaesthesia. The gastro-enteric transit of the six capsules was monitored simultaneously using the temperature and pH information transmitted every 15 s by the capsules (Fig. 2). When it was estimated that the capsules had reached the final section of the ileum (based on pH values ranging between 8 and 9 for approximately 4 hours, Table 2), the capsules were remotely actuated to release their BCG payload. Rectal excretion of the capsule was indicated by a sharp decrease of transmitted temperature to approximately 10 °C; recovery of the capsule was then attempted from latrines.

**Table 2:** Whole Gut Transit Time (WGTT) in individual Group A animals, subdivided into the transit durations in the stomach (Gastric Residence Time, GRT), small intestine (Small Bowel Transit Time, SBTT), and large intestine (Colon Transit Time, CTT)

Animal ID	GRT	SBTT	CTT	WGTT	Delivery area
7D63	12:27	05:31	14:56	32:56:00	Ileum
9377	12:53	09:52	NR	NR	Ileum
A482	03:04	06:38	22:02	31:46	Ileum
903E	13:02	05:31	15:28	34:03	Ileum
9A39	10:07	04:25	18:34	33:07	Ileum
76C6	15:27	03:04	39:09	57:41	Colon
Group results					
Average	11:10	05:50	22:02	37:54	
SD	04:18	02:18	09:58	11:05	
Min	03:04	3:04 1	04:56	31:46	
Max	15:02	7 9:52	39:09	57:04	

WGTT or CTT were not calculated for badger 9377 due to battery exhaustion before excretion (“Not Recorded”, NR). Time expressed in hr.:min format

### Direct vaccination to oropharyngeal mucosa and tonsils

Differences in vaccination protocols between groups are summarised in Table 1. Groups B, C and D badgers received 200 μl BCG on the oral mucosal surface. BCG was applied directly onto the left and right pharyngeal tonsils and under the tongue on the left and right sides of group B badgers (50 μl at each location, 200 μl in total) or onto the left and right tonsils only of groups C and D badgers (100 μl at each location, 200 μl in total) using a micropipette. In addition, in groups C and D, molten lipid (at 37 °C) was also placed onto the surface of the tonsils and sublingual mucosa (200 μl on each mucosal surface for a total of 800 μl) immediately after the application of BCG. BCG was not mixed with the lipids because of the European patent EP 1420818 B1 covering TB vaccines homogeneously dispersed in lipids. The lipids were molten hydrogenated peanut oil (HPO) (CAS Number: 68425–36-5, Sigma-Aldrich) for group C and cocoa butter (CB) (CAS number: 8002-31-1, Parchem, USA) for group D. Badgers were monitored closely for approximately 5 minutes after vaccination for coughing, signs of discomfort or liquid drainage from the mouth, and the animals were then transported to their wooden setts to recover from general anaesthesia.

### Immunological responses

*Interferon gamma release assay (IGRA).* Heparinised whole blood diluted in RPMI medium (Fisher scientific®, Loughborough, UK) supplemented with sodium heparin (Roche®, Basel, Switzerland) and Penicillin/streptomycin (Fisher) was stimulated with a final concentration 30 μg/ml bovine (PPD-B) and avium (PPD-A) tuberculins separately (Prionics, Lelystad, The Netherlands), with pokeweed mitogen (Sigma-Aldrich) at a concentration of 5 μg/ml or without antigen (RPMI), in 1.5 ml aliquots at 37 °C and 5% CO_2_. Supernatants were collected after 16 h stimulation in duplicated aliquots (250μl) and stored at − 80 °C until sandwich ELISA was conducted for IFN-γ levels using anti-badger IFN-γ capture monoclonal antibodies 10H6-C1 and biotinylated-11B9 (badger Interferon Gamma Release Assay, IGRA) [41]. Responses for BCG by IGRA were expressed as Net OD values for PPD-B. The cut-off point of 0.044 for Net PPD-B minus PPD-A, or just Net PPD-B for two badgers in group A and two in group B for which the PPD-A OD values were not available, was used to provide evidence that the badgers were not *M. bovis* infected pre-vaccination [41].

*ELISPOT*. In groups A and B only for practical reasons (the assay was not available for groups C and D), a direct ELISPOT assay was also conducted with isolated peripheral blood mononuclear cells (PBMCs) diluted in RPMI medium supplemented with sodium heparin (Wockhardt UK Ltd) and Penicillin/streptomycin (with 2 × 10^5^ cells per 200 μl in each of duplicate wells). The PBMCs were stimulated with PPD-B and PPD-A, mitogen Concanavalin A (Sigma-Aldrich) at a concentration of 5 μg/ml or no antigen (supplemented RPMI) for 16–20 h in wells pre-coated with monoclonal antibody 10H6-C1 in carbonate buffer. Following incubation, the IFN-γ-producing cells were detected at the bottom of the wells with biotinylated monoclonal antibody 11B9 [42]. The ELISPOT results were expressed as number of Spot Forming Unit (SFU)/million cells.

### Environmental samples collected for culture and PCR

Faecal samples from groups A and B were collected from latrines for analysis by culture/RT-PCR 6 days after vaccination. They were stored frozen at − 80 °C until they were processed for culture and RT-PCR. These samples were added to the collection plans after BCG presence in tissues was successfully demonstrated in groups C and D (faeces were not collected in these groups).

### Collection of tissues post-mortem

At post-mortem examination, tissues were collected using separate sterile sets of instruments and tubes for each tissue: tonsils, left and right retro-pharyngeal and mandibular lymph nodes, left and right parotid gland, spleen, liver, mesenteric and hepatic lymph nodes, gastric fundus, three sections of duodenum, jejunum, ileum, colon and rectum (approximately 2 cm long), pooled thoracic lymph nodes and embryos if present. Tracheal medial section, proximal oesophagus section, urine, and faeces from the rectum were also collected from groups A and B only, as an addition to the sampling plans used for groups C and D. Sections (approximately two-thirds) of the tissues for groups A and B and complete tissues for groups C and D were submitted for culture and PCR testing and weighed. The remaining third of the tissues for groups A and B only was collected into 10% buffered formalin for histological analysis as an addition to the sampling plans used for groups C and D. The left and right tonsil tissues (tonsils, retro-pharyngeal and mandibular lymph nodes) were pooled for groups C and D but collected separately for groups A and B (Table 1). The sampling protocol for study 1 evolved from study 2, following the successful detection of BCG in study 2 when tissues were fully submitted for culture.

### Culture

Homogenisation of tissues and of complete foetuses was conducted in sterile tubes (IKA®, BMT-20-S, Wilmington, USA) for 1–2 min in 3, 5 or 10 ml saline depending on the size of the tissue in groups A and B (0–0.5 g, 0.5–1 g and > 1 g respectively) or with 10 ml saline for all tissues in groups C and D (Table 1). Faecal samples were decontaminated with 4% NaOH (w/v) for 15 min at 37+/− 2 °C, and neutralised with 10% H_2_SO_4_ (v/v)_._ All samples were inoculated onto Modified 7H11 medium (BD Difco™ Mycobacteria 7H11 Agar, BD Biosciences, New Jersey, USA).

Washes of tracheal section (~ 1 cm) in saline, homogenised tissues, urine and treated faeces were each inoculated onto four Modified 7H11 agar (400 μl per plate for groups A and B and 100 μl per plate for groups C and D) for twelve weeks at 37 °C. Solutions in which the Intellicap capsules had been immersed before and after BCG in vitro release were inoculated onto two Modified 7H11 agar plates each (100 μl per plate) and incubated at 37 °C for 4 weeks. BCG was confirmed by spoligotyping of bacterial colonies as described in [43] and RT-PCR. BCG recovery from tissues was expressed as the number of CFU per gram of tissue before homogenisation. To provide an estimation of the total bacterial level per animal, the CFU per gram of tissue were summed and log_10_ transformed.

### Real time (RT)-PCR

DNA was extracted from bacterial colonies growing on solid culture medium and from homogenized tissues, faeces and tracheal wash using the High Pure PCR preparation template kit (Roche Molecular Systems). The primers and probes used in this study are described in Table 3. Positive detection of BCG was reported on bacterial colonies on the basis of a positive response for IS*1081* or IS*6110* (*Mycobacterium tuberculosis* complex), and for IS*1561’* [44] (Table 1 and Additional file 1: Table S1). In tissues, positive detection of BCG was reported on the basis of a positive response for IS*1081,* IS*1561’*, or for RD1 flanking region (in groups A and B only). Tissue samples from Groups A and B were also tested for *hsp65* for mycobacterial species not necessarily belonging to the *M. tuberculosis* complex and for IS*1245* for *M. avium* infection. Reactions were carried out in a 25 μl reaction mix containing TaqMan™ Fast Advanced Master Mix (ThermoFisher Scientific, Villebon sur Yvette, France), 300 nM forward and reverse primers, 250 nM probes, sterile water, and 5 μl of DNA template. Thermocycling conditions were 50 °C for 2 min (1 cycle), followed by one cycle of 20 s at 95 °C and 40 cycles of 3 s at 95 °C and 30 s at 60 °C. PCR inhibition was tested (Diagenode, Thermo Fisher, USA). The negative controls included TE-4 Buffer (10 mM Tris, 0.1 mM EDTA**)** and positive controls were bovine tissue spiked with BCG for each badger and *M. bovis* DNA (wild type strain, spoligotype SB0134) for each PCR series.

**Table 3:** Primers and probes oligonucleotides for real-time-PCR assays

Targeted genes or sequences	Primers-Probe name	Sequence 5′ – 3′	Origin
IS*6110*	TR IS*6110* F	GGT AGC AGA CCT CAC CTA TGT GT	LNR, ANSES
	TR IS*6110* R	AGG CGT CGG TGA CAA AGG	LNR, ANSES
	TR IS*6110* P	(FAM)-CAC GTA GGC GAA CCC-(MGB-NFQ)	LNR, ANSES
IS*1081*	TR IS*1081* F	CCG CCA CCG TGA TTT CGA	LNR, ANSES
	TR IS*1081* R	GCC AGT CCG GGA AAT AGC T	LNR, ANSES
	TR IS*1081* P	(FAM)-CCG CAA CCA TCG ACG TC-(MGB-NFQ)	LNR, ANSES
IS*1245*	TR IS*1245* F	GCC GCC GAA ACG ATC TAC	LNR, ANSES
	TR IS*1245* R	TGA CCC GGT GCG CAG CTT	LNR, ANSES
	TR IS*1245* P	(FAM)-TCG CGT CCG CGC ACG CTG TCC A-(BHQ1)	LNR, ANSES
Hsp65	F MSP	GCC AAG GAG GTC GAG ACC AA	LNR, ANSES
	R MSP	CTC CTC GAC GGT GAT GAC	
	P MSP	(FAM)-ACC TTG TCC ATC GCC TCG GCG AT-(BHQ1)	
RD1 flanking region (BCG)	RD1 F	TAC GCT CGC GTT CGT GGT	LNR, ANSES
	RD1 R	GAT GAG TAT TAC CAG GCC GAC	
	S RD1	(FAM)-TCC GGG CGG CTG GGT GAT GTG -(BHQ1)	
IS*1561’*	TR IS*1561’* F	GAT CCA GGC CGA GAG AAT CTG	[44]
	TR IS*1561’* R	GGA CAA AAG CTT CGC CAA AA	
	TR IS*1561’* P	(FAM)- ACG GCG TTG ATC CGA TTC CGC-(BHQ1)	
Rv1510	Rv1510 F	CCA CGA CTA TGA CTA GGA CAG CAA	[45]
	Rv1510 R	AAG AAC TAT CAA TCG GGC AAG ATC	
	Rv1510 S	(FAM)- ACC AGT GAG GAA ACC-(MGB-NFQ)	

### Histopathology and laser capture microdissection

In groups A and B, each formalin fixed tissue was processed and prepared with Haematoxylin and Eosin (H&E) and Ziehl-Neelsen stained for Acid Fast Bacilli (AFB) identification. Four μm tissue sections were examined under light microscopy (Eclipse Ci. Nikon Instrument UK., Kinston Upon Thames, UK) to ascertain the presence of granulomatous lesions and AFB respectively.

When observed, granulomatous lesions were dissected with a laser-capture micro dissector (Leica LMD6500, Leica Microsystems, Wetzlar, Germany), as previously described [46], in order to explore if BCG could be detected in association with organised granulomatous lesions. Total DNA was extracted using the RecoverAll™ Total Nucleic Acid Isolation Kit (Life technologies, Carlsbad, CA 92008 USA) according to the manufacturer’s protocol and stored at -80 °C until further PCR analysis.

## Results

### Suitability of Medimetrics IntellicapFR® capsules for delivering targeted live vaccine in the ileum of captive badgers

In this study, Medimetrics IntellicapFR® capsules were used for the first time to deliver a live vaccine in vivo*.* It was confirmed in vitro that the dose of live BCG was not compromised during 20 h storage in the reservoir of six IntellicapFR® capsules (data not shown).

Six fresh capsules were used for vaccinating badgers. During their transit through the stomach and digestive tract of group A badgers, the capsules transmitted pH and body temperature successfully (e.g. for badger 7D63 in Fig. 2), which allowed the estimation of transit times (Table 2). No adverse clinical effects were observed in association with the delivery and transit of the capsules. Five out of six capsules were considered successfully actuated in the targeted distal part of the ileum and one capsule appeared to have passed the ileocecal transition before actuation due to the shorter than average small bowel transit time of badger 76C6 (Table 2). Four excreted capsules were recovered from latrines and their opened lids confirmed successful actuation. It was assumed that the two non-recovered capsules were also successfully actuated.

### Culture and RT-PCR for BCG and other mycobacteria

In group A badgers, BCG was mostly recovered from the gut sections and mesenteric lymph node (Tables 4 and 5).

**Table 4:**
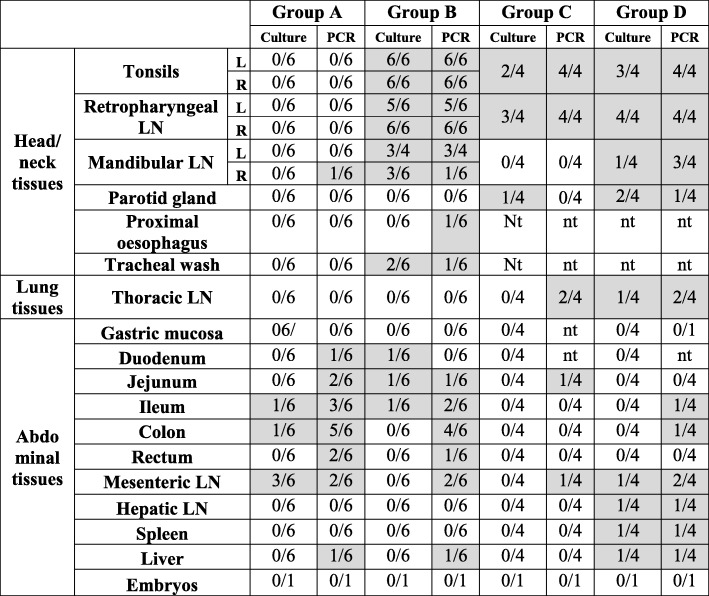
Recovery of BCG by culture or detection by PCR from tissues summarised for groups A-D badgers

Greyed areas indicate when some animals of the groups are positive for BCG detection (“nt” is for “not taken”)

**Table 5:**
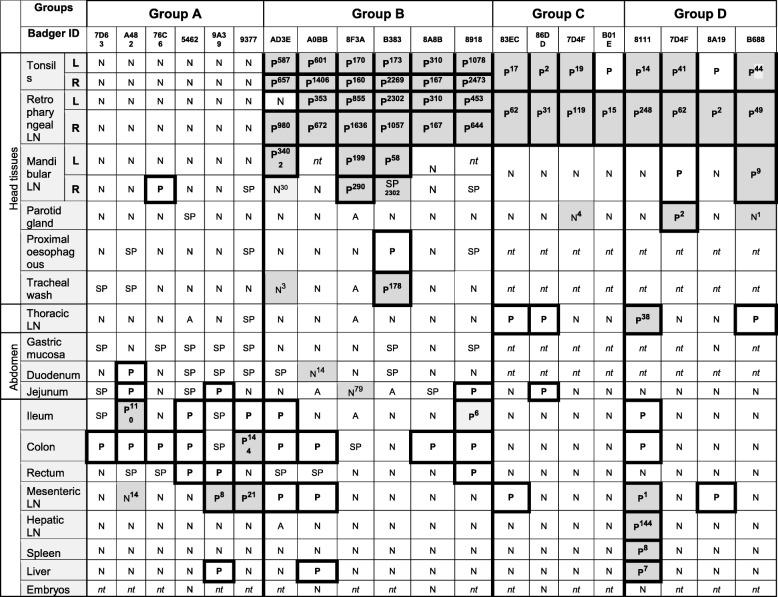
RT- PCR and culture results in tissues for individual badgers in groups A-D

“P” corresponds to positive for BCG, based on positive response for IS*1081,* for RD1 flanking region (in groups A and B only), or IS*1561’*. “SP” is for positive for *hsp65* only and *“*A” is for Cp avium when positive for IS*1245* (in groups A and B only). “N” is for complete negative. Tissue samples from Groups A and B were also tested for *hsp65* for mycobacterial species not necessarily belonging to the M. tb complex and for *M. avium* infection. Greyed areas indicate when live BCG was detected by culture of tissues, with number of cfu/g tissue shown as superscript. “nt” is for “not taken”

Live BCG was recovered by culture from the ileum (in 1 out of 6 animals), the duodenum (1/6), the colon (1/6) and from the mesenteric lymph node (3/6). BCG DNA was detected by RT-PCR in a larger range of tissues and in more animals than by culture: duodenum (1/6), jejunum (2/6), ileum (3/6), colon (5/6), rectum (2/6), mesenteric lymph node (2/6). Badger 76C6, which had been vaccinated in the colon, was PCR-positive (but culture negative) for BCG in each of the three colon sections tested while the other animals generally only had one colon section positive (data not shown). BCG was also detected by PCR in the mandibular lymph node of this animal. Only one badger harboured BCG DNA in the liver (no detection of live BCG by culture). Spleen and hepatic lymph node samples were negative in all group A animals as determined by culture and PCR.

In badgers vaccinated in the oropharyngeal cavity (groups B, C and D), BCG was recovered by culture and detected by PCR from the tonsils and retro-pharyngeal lymph nodes of most of the animals, with both sides infected simultaneously (when tested in group B) (Tables 4 and 5). The drainage of BCG to the mandibular lymph nodes was more variable (in only three out of six animals). Live BCG was recovered from the oesophagus and trachea of some animals (only tested in group B). Drainage to the thoracic lymph nodes was only demonstrated in two group C animals and two group D animals, including by culture in one of them.

**Fig. 3:**
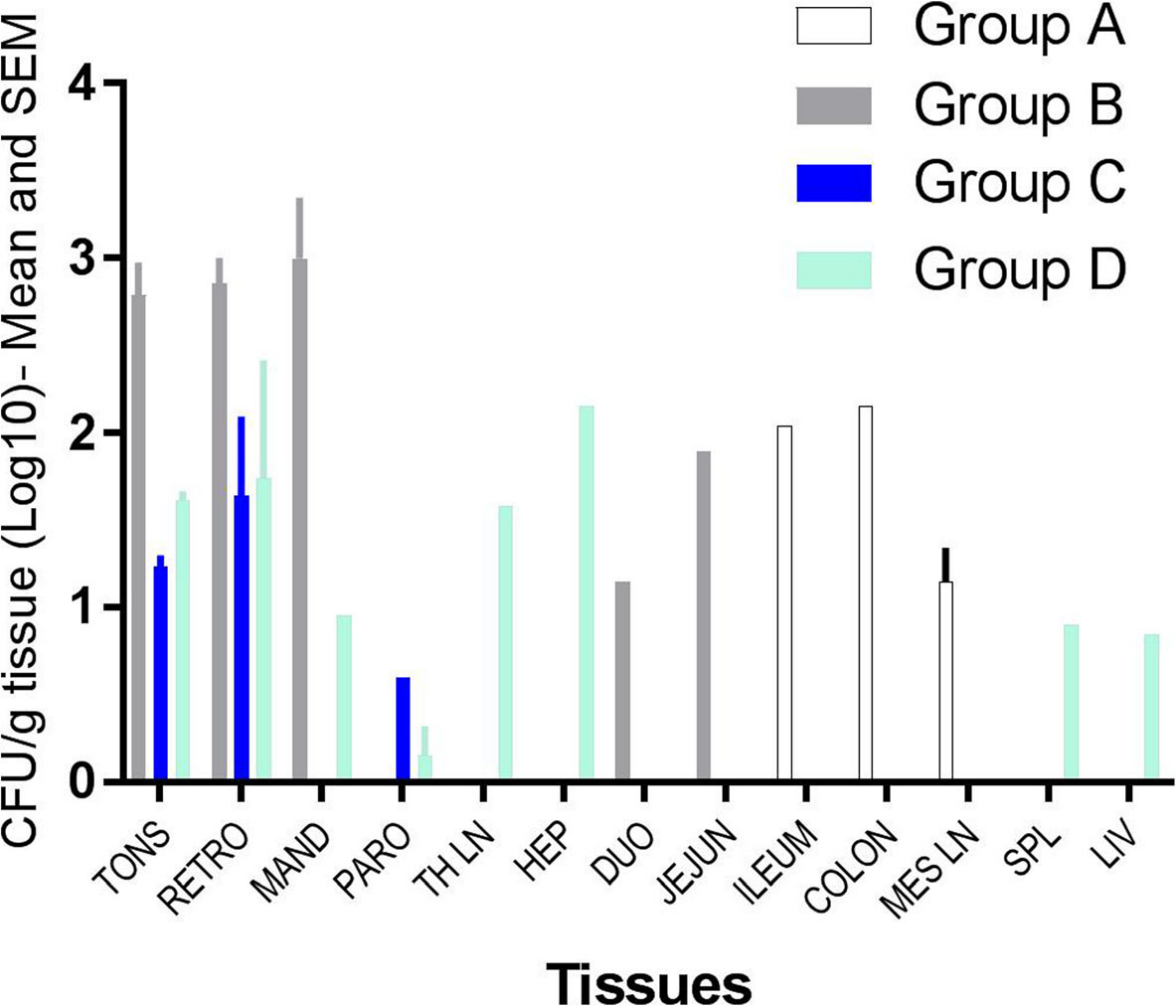
BCG load in tissues by culture as an average for each treatment group. BCG load is given in log_10_ cfu/g tissues. Tissues are listed as in Table 5

Overall, the detection of BCG was more successful by RT-PCR than by culture (36 additional tissues were positive by RT-PCR than by culture), especially in digestive tissues (oesophagus, gastric mucosa, intestinal mucosa). Tissues positive by culture only tended to present low bacterial counts (Table 5). BCG DNA was detected in faeces collected from the latrines of groups A and B six days following vaccination (data not shown) and in the rectum content of one group A badger, 8 weeks after vaccination (badger 7D63), but not live BCG. BCG was not detected in any of the foetuses or urine samples by culture or PCR. The highest average bacterial loads (expressed as CFU/g tissue) were recorded for tonsils, retro-pharyngeal, mandibular, and also hepatic lymph node (in one animal) (Fig. 3). One group D badger (8111) presented an unusually widespread distribution of BCG (Table 5), possibly the consequence of immune compromise caused by a lymphoma (diagnosed post-mortem). Non-specific mycobacteria were identified by PCR in the oral, intestinal tissues and pleura of all groups. DNA for *M. avium hominissuis* was detected in the parotid gland, tracheal tissue, thoracic and hepatic lymph nodes and the gut from one group A and four group B animals (Tables 4 and 5).

### Histopathology

Histopathological lesions in tissues from which BCG was recovered were rare and mild. No AFBs were observed in any samples. In the mesenteric lymph node of one group A badger, small granulomas were detected with only epithelioid macrophages, a few neutrophils, lymphocytes, and a mild cellular necrosis. BCG DNA was detected by PCR after extraction by laser capture microdissection. Live BCG was also cultured from this homogenised tissue. The retro-pharyngeal and mandibular lymph nodes of four group B badgers contained mild granulomatous inflammation with no evidence of mycobacterial infection detected by culture of histology (data not shown).

### Immunology

**Fig. 4:**
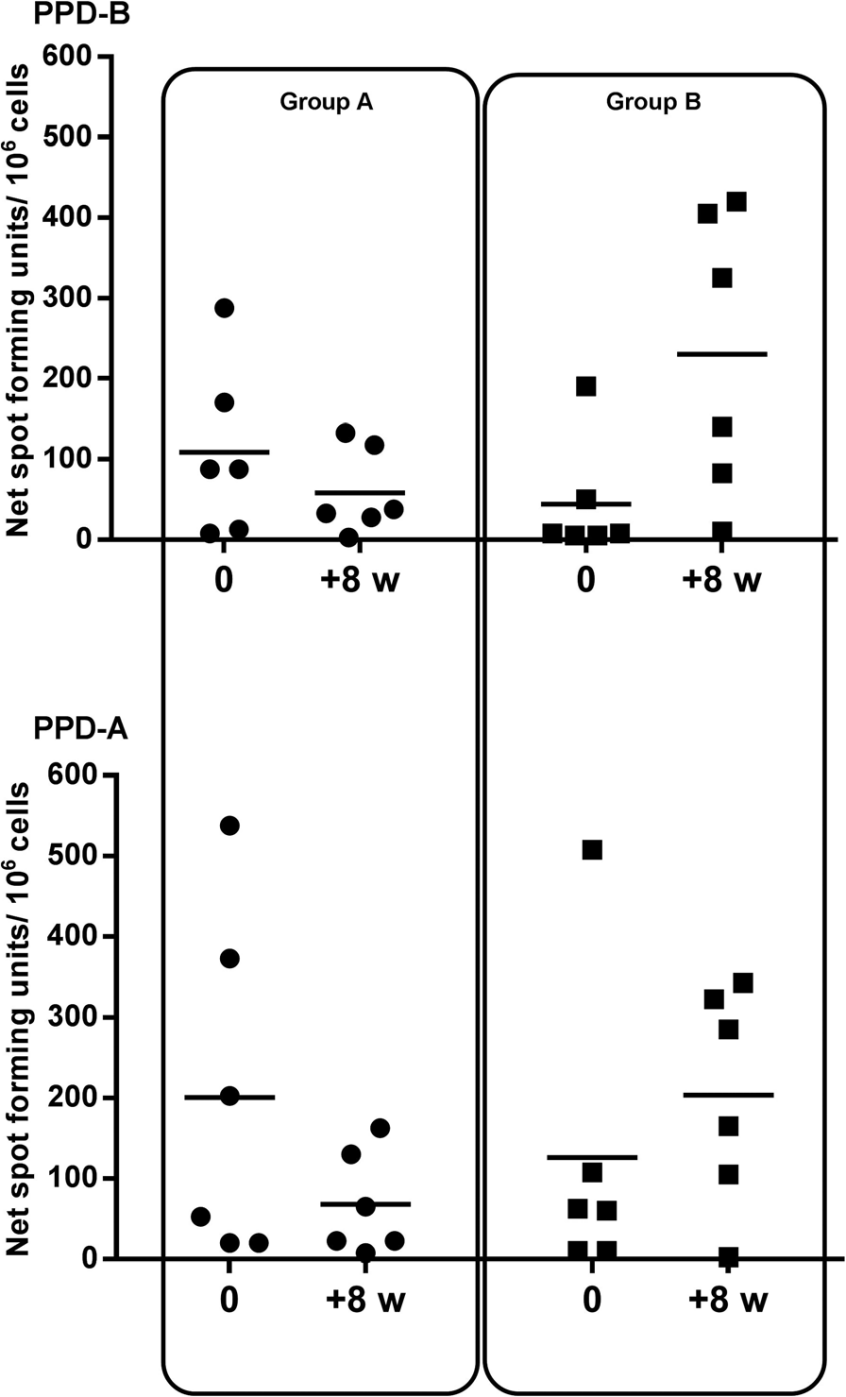
ELISPOT responses before and after vaccination. Measured as Net Spot Forming Units (per million cells) before vaccination and 8 weeks after vaccination for groups A (circles) and B (squares), in response to the antigens bovine tuberculin (PPD-B) and avian tuberculin (PPD-A)

**Fig. 5:**
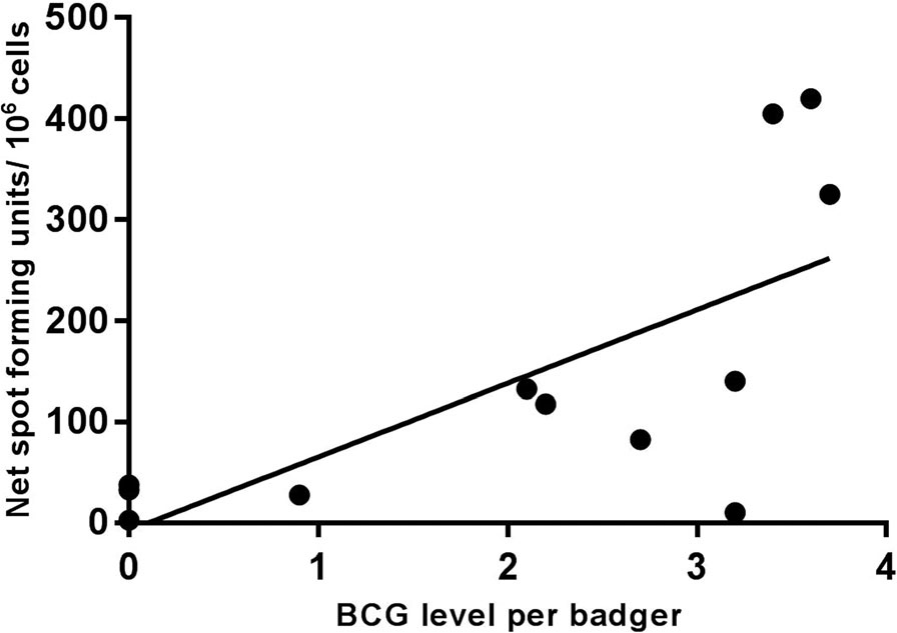
Correlation between the production of IFN-γ and the level of live BCG, 8 weeks after vaccination. The production of IFN-γ was measured by ELISPOT as Net Spot Forming Units (per million cells) and the level of live BCG recovered per group A and B badger from tissues was the log_10_ value of the total CFU per gram of tissue recovered in each individual animal, 8 weeks after vaccination (Pearson r = 0.7038, *p* = 0.0106)

All the badgers were negative by IGRA in whole blood at the time of vaccination (data not shown), which confirmed their TB-free status before the start of the study. The strong response to PPD-B by ELISPOT pre-vaccination in one animal (Fig. 4) was associated with an equally strong response to PPD-A which was not maintained post-vaccination. Post-vaccination, the group B animals had a significant increase in PPD-B responses measured by ELISPOT (Fig. 4) (Mann-Whitney, *p* = 0.0238), also seen by IGRA (data not shown). The overall ELISPOT responses to PPD-B 8 weeks post-vaccination were correlated with the total bacterial score for BCG at the individual level (Pearson r = 0.69, *p* = 0.0139) (Fig. 5).

Live *M. avium* was recovered from the tissues of the two badgers with the strongest responses to PPD-A by ELISPOT pre-vaccination (7D63 - group A and 8918 – group B) and by ELISA in groups C and D (data not shown).

## Discussion

Oral BCG vaccination confers protection to badgers against *M. bovis* infection both experimentally and in the field [21–24]. There is evidence that the persistence of live BCG within the host, even at limited levels, is important for maintaining protection against TB, at least in mice [29–31]. However, in a previous study, live BCG was recovered infrequently from the tissues of orally vaccinated badgers [21], possibly because the sensitivity of detection may have been reduced by the time-interval following vaccination and infection with virulent *M. bovis* (at least 25 weeks). The use of growth media to select for BCG over *M. bovis* did not increase the recovery rate of BCG [23]. As vaccine dose and method of delivery (e.g. targeting specific parts of the gut associated mucosa and use of gastro-protective agents) affects the persistence of live BCG [35, 47, 48], we sought a better understanding of mucosal uptake and immunogenicity of BCG in badgers.

In these studies, we show that BCG delivered orally can persist within badger tissues and stimulate the production of IFN-γ by PBMC and whole blood 8 weeks later. Persistence and immune responses measured were greater in animals after vaccination by oropharyngeal instillation followed by drainage in the oesophagus compared with those vaccinated directly in the ileum.

The IntellicapFR® capsules allowed a defined dose of live BCG to be targeted directly to the ileum. This anatomical area would have been difficult to target by surgery or endoscopy because of the lack of clear anatomical features in badgers (the ileocaecal valve is absent), whereas the change in pH profiles transmitted by the capsules permitted simple identification of the transition between the small and the large intestine. This is also an important refinement in terms of the 3Rs ethos of animal experimentation [49]. In addition, the use of the capsules allowed us to establish that badgers have a longer gastric residence time (GRT) (average of 11 h) than mice [50], possum [51] or patients fitted with Intellicap devices [32], possibly because the animals were not fasted [52], because of the winter season [53] or of a residual effect of the anaesthetic drugs on gastro-enteric motility [54]. Gastric and intestinal transit time/pH data peculiar to the target species are useful for developing an effective oral vaccine.

Using Intellicap® capsules, live BCG was delivered directly in the distal ileum, presumably the most favourable site for bacterial uptake given the highest densities of the gut specialised immunological inductive sites (Peyer’s patches) in badgers are seen there (Nunez, unpublished data). *M. bovis* can be expected to drain from the enteric lumen to the mesenteric lymph nodes in badgers as in other mustelids [55, 56] and mice [57–59], although variable drainage levels [60] and kinetics [30, 61] are possible. However, live BCG was recovered inconsistently and at low levels from the mesenteric lymph nodes and other gut tissues (at least on the basis of positive PCR results). It cannot be excluded that live BCG would be absorbed more efficiently at other intestinal level than the ileum, although this was not suggested by the data obtained when BCG was delivered in the oropharynx; in this group, BCG was only detected at small level in the duodenum and the jejunum as well as in the ileum, the colon, and the mesenteric lymph node.

The concentration of live BCG per gram of homogenised tissue was highest in the tonsils, retro-pharyngeal, mandibular, and hepatic lymph nodes of badgers vaccinated directly in the oropharyngeal cavity and at levels consistent to those found in other species (mice and guinea-pigs) vaccinated with BCG doses ranging from 10^6^ to 10^7^ CFUs [34–36, 57, 58, 62]. Evaluations of the BCG dose actually absorbed by each badger based on total levels recovered from tissues suggest that only a fraction of the dose delivered was actually absorbed and retained viability, as expected from previously reported work [60]. The differences in protocol between studies make direct quantitative comparisons between A-B and C-D difficult. The relatively higher level of BCG recovered from mandibular lymph nodes of group B animals may be associated with additional sublingual uptake in this group [63].

Lipid-formulated BCG has given significant protection against virulent TB challenge in mice [34, 35, 64], guinea pigs [36], cattle [37], deer [38], and badgers [21–24]. Evidence for the role of lipids in enhancing uptake and tissue persistence and the immunogenicity of oral BCG compared with unformulated BCG has been reported in mice [34, 58], with the suggestion that lipids may protect BCG against gastric and enteric degradation. Two different lipids were used with BCG to assess whether they would contribute to a higher level of BCG uptake and persistence. HPO is the holding matrix for BCG in the vaccine bait developed for badgers [26]. CB was considered an alternative to HPO with the potential for better mucosal uptake as it becomes molten more rapidly after consumption than HPO at body temperature (38–39 °C in healthy badgers [65]) with a melting point of 34 °C instead of 39 °C. However, the results of the present study do not suggest an overall significant increase in mucosal persistence of BCG when molten lipids are added, although some evidence of better pulmonary drainage to the thoracic lymph nodes were obtained which may be significant for inducing local pulmonary protection [18]. A correlation between the strength of the Th1 immune responses in thoracic lymph nodes 16 and 24 days post-infection and the containment of *M. tuberculosis* multiplication in the lung has been observed in infected mice [66, 67].

We measured the production of IFN-γ by peripheral blood cells, an immunological marker of successful BCG delivery and uptake in animals [35, 68–70]. The production of peripheral IFN-γ following vaccination is critical for the development of protective responses against TB [71–73], and although it does not directly correlate with protection [56, 69, 74, 75], it reflects the level of persisting live BCG (at least in mice) [29, 30, 35, 76]. In the present study, IFN-γ production in response to PPD-B was correlated with the quantitative estimation of live BCG per badger (greatest responses were seen in animals vaccinated by oropharyngeal instillation). The high responses to avian tuberculin (PPD-A) by some badgers were not unexpected given that environmental mycobacteria, including *M. avium*, were recovered from these animals, and mycobacteria not belonging to the *M. tuberculosis* complex were detected in tissues. The impact of this co-infection on potentially reducing, or enhancing, the development of protective immunological responses in badgers is unknown but could be important [77–84].

In this study, the delivery of oral BCG was safe, with no remarkable pathology associated with the presence of live BCG in tissues. More severe granulomatous lesions had been observed after subcutaneous BCG vaccination [42]. One mild granulomatous lymph node lesion contained BCG DNA. The widespread recovery of live BCG from one animal was associated with a lymphoma which may have increased its susceptibility to BCG. No BCG presence was observed in any foetus of vaccinated mothers. Infections with *Histoplasma capsulatum* [85, 86] and *Emmonsia crescens* [87, 88] were detected in two animals in this study. They are common in badgers and were not associated with any unusual BCG spread or persistence, nor unusual immune responses.

In all groups, a larger proportion of tissues were positive by RT-PCR than by culture, with BCG-specific amplification using the RD1 flanking region primers in parallel with IS*6110* or IS*1081* primers. BCG DNA may have been detected from either intact bacterial cells (of unknown viability) or free within the tissue following the uptake of dying or dead bacteria by lymphoid tissues. In the present study, no live BCG was cultured from faeces samples of vaccinated badgers, either from latrines six days after vaccination or from rectal samples collected at post-mortem 8 weeks after vaccination. However, BCG DNA was detected in faeces, showing some level of faecal excretion but without information regarding the bacterial viability. Previous data also showed infrequent recovery of live BCG from badger faeces after oral vaccination [21, 23], even with a vaccine dose exceeding 10^9^ CFU [89]. This low level of live BCG excretion from badgers, also reported in other mustelids such as ferrets (*Mustela furo*) [56], contrasts with the relatively high numbers of BCG recovered from the faeces of vaccinated possums [59] and mice [90]. It suggests that mustelids may have a particularly hostile gastro-enteric environment for *M. bovis* (both attenuated BCG and virulent *M. bovis* [21, 87, 91]), which needs to be taken into account when developing a live oral vaccine for use in badgers. Indeed, ferrets vaccinated with a BCG suspension (5 × 10^7^ CFU) directly in the duodenum [74] were less efficiently protected than possums vaccinated by the same route and with similar vaccine dose and formulation [92]. Inactivation of live BCG would be expected when exposed to gastric acidity, bile and pancreatic secretions [60] and possibly local bacterial flora [93, 94].

Based on our data, strategies for delivering live BCG to mustelids should therefore consider enteric protection and/or targeting pre-gastric immune tissues, for example, the tonsils [95] and naso-pharynx. These tissues, as well as the gut [58, 96, 97] and the lungs [98], possess Peyer’s patches with M cells in particular that are permissive to bacteria (including BCG) and mediate the induction of the adaptive immune responses [99]. M cells therefore constitute interesting targets for mucosal vaccines [100–102]. Efficient oral delivery systems could include muco-adhesive systems directly incorporated in the baits, or larger volumes of vaccines.

## Conclusion

It is generally accepted that the dose, viability and persistence in the host of BCG are crucial for lasting vaccine protection [29], underpinning the efficient uptake of live BCG by the targeted mucosal surfaces. The rational development of an oral vaccine against TB in badgers should therefore address these points.

In the present study, we demonstrate that BCG administered orally can efficiently penetrate and persist in tonsils, drain to lymph nodes and stimulate the production of IFN-γ by peripheral blood cells in badgers for at least 8 weeks. In contrast, direct enteric delivery of BCG failed to achieve consistent drainage of live BCG to the mesenteric lymph nodes and to stimulate strong immune responses. Together these data suggest that vaccine baits should aim to release BCG in the oropharyngeal region or that it may be necessary to protect BCG from enteric degradation in order to achieve optimal protective efficacy. This hypothesis would require confirmation by testing the protective efficacy of BCG delivered in the gut versus the mouth. Oral vaccination with BCG in the context of molten lipids is already known to be protective [23], but our study indicates that it may be beneficial for the colonisation of the trachea and bronchia by live BCG. This protection could potentially be improved upon and/or the vaccine dose reduced once key parameters for optimal mucosal uptake and stimulation of pulmonary immune responses are identified.

## Additional file

**Additional file 1: Table S1.** Primers used for the detection of mycobacteria by RT-PCR

## Data Availability

The dataset supporting the conclusions of this article are available if requested.

## Abbreviations

AFB: Acid Fast Bacilli
BCG: Bacille of Calmette and Guerin
CB: Cocoa butter
CTT: Colon Transit Time
GRT: Gastric Residence Time
H&E: Haematoxylin and Eosin
HPO: Hydrogenated peanut oil
IFN-γ: Interferon gamma
IGRA: Interferon gamma release assay
IR: Infra-red
*M. sp*: Non tuberculous Mycobacterium
NR: Not Recorded
nt: not taken
SBTT: Small Bowel Transit Time
TB: Tuberculosis
WGTT: Whole Gut Transit Time

## References

[1] Thiermann AB (2015). International standards: the world organisation for animal health terrestrial animal health code. Rev Sci Tech.

[2] Palmer MV (2013). *Mycobacterium bovis*: characteristics of wildlife reservoir hosts. Transbound Emerg Dis.

[3] Gormley E, Corner L (2009). Control of TB in wildlife by oral BCG vaccination. Expert review of vaccines.

[4] Wilson GJ, Carter SP, Delahay RJ (2011). Advances and prospects for management of TB transmission between badgers and cattle. Vet Microbiol.

[5] Balseiro A, Gonzalez-Quiros P, Rodriguez O, Francisca Copano M, Merediz I, de Juan L, Chambers MA, Delahay RJ, Marreros N, Royo LJ (2013). Spatial relationships between Eurasian badgers (*Meles meles*) and cattle infected with *Mycobacterium bovis* in northern Spain. Vet J.

[6] Payne A, Boschiroli ML, Gueneau E, Moyen JL, Rambaud T, Dufour B, Gilot-Fromont E, Hars J (2012). Bovine tuberculosis in “Eurasian” badgers (Meles meles) in France. Eur J Wildl Res.

[7] Carter SP, Delahay RJ, Smith GC, Macdonald DW, Riordan P, Etherington TR, Pimley ER, Walker NJ, Cheeseman CL (2007). Culling-induced social perturbation in Eurasian badgers *Meles meles* and the management of TB in cattle: an analysis of a critical problem in applied ecology. Proc Biol Sci.

[8] Fine PEM, Carneiro IAM, Milstien JB, Clements CJ (1999). Issues relating to the use of BCG in immunization programmes.

[9] Cross ML, Buddle BM, Aldwell FE (2007). The potential of oral vaccines for disease control in wildlife species. Vet J.

[10] Calmette A (1931). Preventive vaccination against tuberculosis with BCG. Proc R Soc Med.

[11] Luca S, Mihaescu T (2013). History of BCG vaccine. Maedica.

[12] Benevolo-de-Andrade TC, Monteiro-Maia R, Cosgrove C, Castello-Branco LR (2005). BCG Moreau Rio de Janeiro: an oral vaccine against tuberculosis--review. Mem Inst Oswaldo Cruz.

[13] Monteiro-Maia R (2014). TdPR: Oral bacillus Calmette-Guérin vaccine against tuberculosis: why not?. Mem Inst Oswaldo Cruz.

[14] Beverley PC, Sridhar S, Lalvani A, Tchilian EZ (2014). Harnessing local and systemic immunity for vaccines against tuberculosis. Mucosal Immunol.

[15] Perdomo C, Zedler U, Kuhl AA, Lozza L, Saikali P, Sander LE, Vogelzang A, Kaufmann SH, Kupz A. Mucosal BCG Vaccination Induces Protective Lung-Resident Memory T Cell Populations against Tuberculosis. MBio. 2016:**7**(6). 10.1128/mBio.01686-16 PMC5120139 27879332

[16] Diogo GR, Reljic R (2014). Development of a new tuberculosis vaccine: is there value in the mucosal approach?. Immunotherapy.

[17] Caetano LA, Almeida AJ, Goncalves LM (2014). Approaches to tuberculosis mucosal vaccine development using nanoparticles and microparticles: a review. J Biomed Nanotechnol.

[18] Chen L, Wang J, Zganiacz A, Xing Z (2004). Single intranasal mucosal Mycobacterium bovis BCG vaccination confers improved protection compared to subcutaneous vaccination against pulmonary tuberculosis. Infect Immun.

[19] Tenland E, Hakansson G, Alaridah N, Lutay N, Ronnholm A, Hallgren O, Westergren-Thorsson G, Godaly G (2016). Innate immune responses after airway epithelial stimulation with *Mycobacterium bovis* Bacille-Calmette Guerin. PLoS One.

[20] Cha SB, Kim WS, Kim JS, Kim H, Kwon KW, Han SJ, Eum SY, Cho SN, Shin SJ (2015). Repeated aerosolized-boosting with gamma-irradiated *Mycobacterium bovis* BCG confers improved pulmonary protection against the Hypervirulent *Mycobacterium tuberculosis* strain HN878 in mice. PLoS One.

[21] Corner LA, Costello E, O'Meara D, Lesellier S, Aldwell FE, Singh M, Hewinson RG, Chambers MA, Gormley E (2010). Oral vaccination of badgers (*Meles meles*) with BCG and protective immunity against endobronchial challenge with *Mycobacterium bovis*. Vaccine.

[22] Murphy D, Costello E, Aldwell FE, Lesellier S, Chambers MA, Fitzsimons T, Corner LA, Gormley E (2014). Oral vaccination of badgers (*Meles meles*) against tuberculosis: comparison of the protection generated by BCG vaccine strains Pasteur and Danish. Vet J.

[23] Chambers MA, Aldwell F, Williams GA, Palmer S, Gowtage S, Ashford R, Dalley DJ, Dave D, Weyer U, Salguero FJ (2017). The effect of Oral vaccination with *Mycobacterium bovis* BCG on the development of tuberculosis in captive European badgers (*Meles meles*). Front Cell Infect Microbiol.

[24] Gormley E, Ni Bhuachalla D, O'Keeffe J, Murphy D, Aldwell FE, Fitzsimons T, Stanley P, Tratalos JA, McGrath G, Fogarty N (2017). Oral vaccination of free-living badgers (*Meles meles*) with Bacille Calmette Guerin (BCG) vaccine confers protection against tuberculosis. PLoS One.

[25] Robertson A, Delahay RJ, McDonald RA, Aylett P, Henderson R, Gowtage S, Chambers MA, Carter SP (2016). Behaviour of European badgers and non-target species towards candidate baits for oral delivery of a tuberculosis vaccine. Prev Vet Med.

[26] Gowtage S, Williams GA, Henderson R, Aylett P, MacMorran D, Palmer S, Robertson A, Lesellier S, Carter SP, Chambers MA (2017). Testing of a palatable bait and compatible vaccine carrier for the oral vaccination of European badgers (*Meles meles*) against tuberculosis. Vaccine.

[27] Lesellier S, Palmer S, Gowtage-Sequiera S, Ashford R, Dalley D, Dave D, Weyer U, Salguero FJ, Nunez A, Crawshaw T (2011). Protection of Eurasian badgers (*Meles meles*) from tuberculosis after intra-muscular vaccination with different doses of BCG. Vaccine.

[28] Holmgren J, Czerkinsky C (2005). Mucosal immunity and vaccines. Nat Med.

[29] Kaveh DA, Carmen Garcia-Pelayo M, Hogarth PJ (2014). Persistent BCG bacilli perpetuate CD4 T effector memory and optimal protection against tuberculosis. Vaccine.

[30] Olsen AW, Brandt L, Agger EM, van Pinxteren LA, Andersen P (2004). The influence of remaining live BCG organisms in vaccinated mice on the maintenance of immunity to tuberculosis. Scand J Immunol.

[31] Orme IM (1988). Induction of nonspecific acquired resistance and delayed-type hypersensitivity, but not specific acquired resistance in mice inoculated with killed mycobacterial vaccines. Infect Immun.

[32] Becker D, Zhang J, Heimbach T, Penland RC, Wanke C, Shimizu J, Kulmatycki K (2014). Novel orally swallowable IntelliCap((R)) device to quantify regional drug absorption in human GI tract using diltiazem as model drug. AAPS PharmSciTech.

[33] Aldwell FE, Cross ML, Fitzpatrick CE, Lambeth MR, de Lisle GW, Buddle BM (2006). Oral delivery of lipid-encapsulated *Mycobacterium bovis* BCG extends survival of the bacillus in vivo and induces a long-term protective immune response against tuberculosis. Vaccine.

[34] Aldwell FE, Baird MA, Fitzpatrick CE, McLellan AD, Cross ML, Lambeth MR, Buchan GS (2005). Oral vaccination of mice with lipid-encapsulated *Mycobacterium bovis* BCG: anatomical sites of bacterial replication and immune activity. Immunol Cell Biol.

[35] Cross ML, Lambeth MR, Coughlan Y, Aldwell FE (2007). Oral vaccination of mice with lipid-encapsulated *Mycobacterium bovis* BCG: effect of reducing or eliminating BCG load on cell-mediated immunity. Vaccine.

[36] Clark S, Cross ML, Nadian A, Vipond J, Court P, Williams A, Hewinson RG, Aldwell FE, Chambers MA (2008). Oral vaccination of Guinea pigs with a Mycobacterium bovis bacillus Calmette-Guerin vaccine in a lipid matrix protects against aerosol infection with virulent M. bovis. Infect Immun.

[37] Buddle BM, Aldwell FE, Skinner MA, de Lisle GW, Denis M, Vordermeier HM, Hewinson RG, Wedlock DN (2005). Effect of oral vaccination of cattle with lipid-formulated BCG on immune responses and protection against bovine tuberculosis. Vaccine.

[38] Nol P, Palmer MV, Waters WR, Aldwell FE, Buddle BM, Triantis JM, Linke LM, Phillips GE, Thacker TC, Rhyan JC (2008). Efficacy of oral and parenteral routes of *Mycobacterium bovis* bacille Calmette-Guerin vaccination against experimental bovine tuberculosis in white-tailed deer (*Odocoileus virginianus*): a feasibility study. J Wildl Dis.

[39] Cavalerie L, Courcoul A, Boschiroli ML, Reveillaud E, Gay P (2015). Tuberculose bovine en France en 2014: une situation stable. Bull Epid Sante Anim Alim.

[40] Kilkenny C, Browne WJ, Cuthill IC, Emerson M, Altman DG (2010). Improving bioscience research reporting: the ARRIVE guidelines for reporting animal research. PLoS Biol.

[41] Dalley D, Dave D, Lesellier S, Palmer S, Crawshaw T, Hewinson RG, Chambers M (2008). Development and evaluation of a gamma-interferon assay for tuberculosis in badgers (*Meles meles*). Tuberculosis.

[42] Lesellier S, Palmer S, Dalley DJ, Dave D, Johnson L, Hewinson RG, Chambers MA (2006). The safety and immunogenicity of Bacillus Calmette-Guerin (BCG) vaccine in European badgers (*Meles meles*). Vet Immunol Immunopathol.

[43] Roring S, Scott A, Brittain D, Walker I, Hewinson G, Neill S, Skuce R (2002). Development of variable-number tandem repeat typing of *Mycobacterium bovis*: comparison of results with those obtained by using existing exact tandem repeats and spoligotyping. J Clin Microbiol.

[44] Barbier E, Boschiroli ML, Gueneau E, Rochelet M, Payne A, de Cruz K, Blieux AL, Fossot C, Hartmann A (2016). First molecular detection of *Mycobacterium bovis* in environmental samples from a French region with endemic bovine tuberculosis. J Appl Microbiol.

[45] Halse TA, Escuyer VE, Musser KA (2011). Evaluation of a single-tube multiplex real-time PCR for differentiation of members of the *Mycobacterium tuberculosis* complex in clinical specimens. J Clin Microbiol.

[46] Aranday-Cortes E, Bull NC, Villarreal-Ramos B, Gough J, Hicks D, Ortiz-Pelaez A, Vordermeier HM, Salguero FJ (2013). Upregulation of IL-17A, CXCL9 and CXCL10 in early-stage granulomas induced by *Mycobacterium bovis* in cattle. Transbound Emerg Dis.

[47] Ancelet LR, Aldwell FE, Rich FJ, Kirman JR (2012). Oral vaccination with lipid-formulated BCG induces a long-lived, multifunctional CD4(+) T cell memory immune response. PLoS One.

[48] Clark S, Cross ML, Smith A, Court P, Vipond J, Nadian A, Hewinson RG, Batchelor HK, Perrie Y, Williams A (2008). Assessment of different formulations of oral *Mycobacterium bovis* Bacille Calmette-Guerin (BCG) vaccine in rodent models for immunogenicity and protection against aerosol challenge with *M. bovis*. Vaccine.

[49] Kirk RGW (2018). Recovering the principles of humane experimental technique: the 3Rs and the human essence of animal research. Sci Technol Hum Values.

[50] Myagmarjalbuu B, Moon MJ, Heo SH, Jeong SI, Park JS, Jun JY, Jeong YY, Kang HK (2013). Establishment of a protocol for determining gastrointestinal transit time in mice using barium and radiopaque markers. Korean J Radiol.

[51] McDowell A, Nicoll JJ, McLeod BJ, Tucker IG, Davies NM (2005). Gastrointestinal transit in the common brushtail possum measured by gamma scintigraphy. Int J Pharm.

[52] Mahar KM, Portelli S, Coatney R, Chen EP (2012). Gastric pH and gastric residence time in fasted and fed conscious beagle dogs using the bravo pH system. J Pharm Sci.

[53] McClune DW, Kostka B, Delahay RJ, Montgomery WI, Marks NJ, Scantlebury DM (2015). Winter is coming: seasonal variation in resting metabolic rate of the European badger (*Meles meles*). PLoS One.

[54] Maugeri S, Ferre JP, Intorre L, Soldani G (1994). Effects of medetomidine on intestinal and colonic motility in the dog. J Vet Pharmacol Ther.

[55] Ragg JR, Waldrup KA, Moller H (1995). The distribution of gross lesions of tuberculosis caused by Mycobacterium bovis in feral ferrets (Mustela furo) from Otago, New Zealand. N Z Vet J.

[56] Qureshi T, Labes RE, Cross ML, Griffin JF, Mackintosh CG (1999). Partial protection against oral challenge with Mycobacterium bovis in ferrets (Mustela furo) following oral vaccination with BCG. The international journal of tuberculosis and lung disease : the official journal of the International Union against Tuberculosis and Lung Disease.

[57] Lagranderie M, Chavarot P, Balazuc AM, Marchal G (2000). Immunogenicity and protective capacity of Mycobacterium bovis BCG after oral or intragastric administration in mice. Vaccine.

[58] Dorer DE, Czepluch W, Lambeth MR, Dunn AC, Reitinger C, Aldwell FE, McLellan AD (2007). Lymphatic tracing and T cell responses following oral vaccination with live *Mycobacterium bovis* (BCG). Cell Microbiol.

[59] Wedlock DN, Aldwell FE, Keen D, Skinner MA, Buddle BM (2005). Oral vaccination of brushtail possums (*Tichosurus vulpecula*) with BCG: immune responses, persistence of BCG in lymphoid organs and excretion in faeces. N Z Vet J.

[60] Mortatti RC, Maia LC, Fonseca LS (1987). Absorption of *Mycobacterium bovis* BCG administered by the oral route. Vaccine.

[61] Macpherson AJ, Smith K (2006). Mesenteric lymph nodes at the center of immune anatomy. J Exp Med.

[62] Cross ML, Lambeth MR, Aldwell FE (2008). Murine cytokine responses following multiple oral immunizations using lipid-formulated mycobacterial antigens. Immunol Cell Biol.

[63] Czerkinsky C, Cuburu N, Kweon MN, Anjuere F, Holmgren J (2011). Sublingual vaccination. Hum Vaccin.

[64] Aldwell FE, Cross ML, Fitzpatrick CE, Lambeth MR, de Lisle GW, Buddle BM: Oral delivery of lipid-encapsulated Mycobacterium bovis BCG extends survival of the bacillus in vivo and induces a long-term protective immune response against tuberculosis. Vaccine 2005. 10.1016/j.vaccine.2005.11.017 16332403

[65] Davison KE, Hughes JM, Gormley E, Lesellier S, Costello E, Corner LA (2007). Evaluation of the anaesthetic effects of combinations of ketamine, medetomidine, romifidine and butorphanol in European badgers (*Meles meles*). Vet Anaesth Analg.

[66] Gallegos AM, Pamer EG, Glickman MS (2008). Delayed protection by ESAT-6-specific effector CD4+ T cells after airborne M. tuberculosis infection. J Exp Med.

[67] Wolf AJ, Desvignes L, Linas B, Banaiee N, Tamura T, Takatsu K, Ernst JD (2008). Initiation of the adaptive immune response to *Mycobacterium tuberculosis* depends on antigen production in the local lymph node, not the lungs. J Exp Med.

[68] Rist N, Canetti G, Boisvert H, Le Lirzin M (1967). The BCG antibiogram. Diagnostic value of resistance to cycloserine. Rev Tuberc Pneumol (Paris).

[69] Buddle BM, Aldwell FE, Keen DL, Parlane NA, Hamel KH, de Lisle GW (2006). Oral vaccination of brushtail possums with BCG: investigation into factors that may influence vaccine efficacy and determination of duration of protection. N Z Vet J.

[70] Buddle BM, Aldwell FE, de Lisle GW, Vordermeier HM, Hewinson RG, Wedlock DN (2011). Low oral BCG doses fail to protect cattle against an experimental challenge with *Mycobacterium bovis*. Tuberculosis.

[71] Flynn JL, Chan J, Triebold KJ, Dalton DK, Stewart TA, Bloom BR (1993). An essential role for interferon gamma in resistance to Mycobacterium tuberculosis infection. J Exp Med.

[72] Cooper AM, Dalton DK, Stewart TA, Griffin JP, Russell DG, Orme IM (1993). Disseminated tuberculosis in interferon gamma gene-disrupted mice. J Exp Med.

[73] Seneviratne SL, Doffinger R, Macfarlane J, Ceron-Gutierrez L, Amel Kashipaz MR, Robbins A, Patel T, Powell PT, Kumararatne DS, Powell RJ (2007). Disseminated *Mycobacterium tuberculosis* infection due to interferon gamma deficiency. Response to replacement therapy. Thorax.

[74] Cross ML, Labes RE, Griffin JF, Mackintosh CG (2000). Systemic but not intra-intestinal vaccination with BCG reduces the severity of tuberculosis infection in ferrets (Mustela furo). The international journal of tuberculosis and lung disease : the official journal of the International Union against Tuberculosis and Lung Disease.

[75] Vordermeier HM, Perez de Val B, Buddle BM, Villarreal-Ramos B, Jones GJ, Hewinson RG, Domingo M (2014). Vaccination of domestic animals against tuberculosis: review of progress and contributions to the field of the TBSTEP project. Res Vet Sci.

[76] Vipond J, Cross ML, Lambeth MR, Clark S, Aldwell FE, Williams A (2008). Immunogenicity of orally-delivered lipid-formulated BCG vaccines and protection against *Mycobacterium tuberculosis* infection. Microbes Infect.

[77] Brandt L, Feino Cunha J, Weinreich Olsen A, Chilima B, Hirsch P, Appelberg R, Andersen P (2002). Failure of the Mycobacterium bovis BCG vaccine: some species of environmental mycobacteria block multiplication of BCG and induction of protective immunity to tuberculosis. Infect Immun.

[78] Palmer CE, Long MW (1966). Effects of infection with atypical mycobacteria on BCG vaccination and tuberculosis. Am Rev Respir Dis.

[79] Stanford JL, Shield MJ, Rook GA (1981). How environmental mycobacteria may predetermine the protective efficacy of BCG. Tubercle.

[80] Buddle BM, Wards BJ, Aldwell FE, Collins DM, de Lisle GW (2002). Influence of sensitisation to environmental mycobacteria on subsequent vaccination against bovine tuberculosis. Vaccine.

[81] Mihu MR, Nosanchuk JD (2012). Histoplasma virulence and host responses. Int J Microbiol.

[82] Balseiro A, Rodriguez O, Gonzalez-Quiros P, Merediz I, Sevilla IA, Dave D, Dalley DJ, Lesellier S, Chambers MA, Bezos J (2011). Infection of Eurasian badgers (*Meles meles*) with *Mycobacterium bovis* and *Mycobacterium avium* complex in Spain. Vet J.

[83] Hernandez-Pando R, Aguilar D, Orozco H, Cortez Y, Brunet LR, Rook GA (2008). Orally administered *Mycobacterium vaccae* modulates expression of immunoregulatory molecules in BALB/c mice with pulmonary tuberculosis. Clin Vaccine Immunol.

[84] Saunders GK, Thomsen BV (2006). Lymphoma and *Mycobacterium avium* infection in a ferret (*Mustela putorius furo*). J Vet Diagn Investig.

[85] Bauder B, Kubber-Heiss A, Steineck T, Kuttin ES, Kaufman L (2000). Granulomatous skin lesions due to histoplasmosis in a badger (*Meles meles*) in Austria. Med Mycol.

[86] Grosse G, Staib F, Rapp J, Rang H, Heise W, Kaufman L (1997). Pathological and epidemiological aspects of skin lesions in histoplasmosis. Observations in an AIDS patient and badgers outside endemic areas of histoplasmosis. Zentralbl Bakteriol.

[87] Corner LA, Murphy D, Gormley E (2011). *Mycobacterium bovis* infection in the Eurasian badger (*Meles meles*): the disease, pathogenesis, epidemiology and control. J Comp Pathol.

[88] Simpson VR, Tomlinson AJ, Stevenson K, McLuckie JA, Benavides J, Dagleish MP (2016). A post-mortem study of respiratory disease in small mustelids in south-West England. BMC Vet Res.

[89] Perrett S, Lesellier S, Rogers F, Williams GA, Gowtage S, Palmer S, Dalley D, Dave D, Weyer U, Wood E (2018). Assessment of the safety of Bacillus Calmette-Guerin vaccine administered orally to badgers (*Meles meles*). Vaccine.

[90] Lagranderie MR, Balazuc AM, Deriaud E, Leclerc CD, Gheorghiu M (1996). Comparison of immune responses of mice immunized with five different Mycobacterium bovis BCG vaccine strains. Infect Immun.

[91] Chambers MA, Rogers F, Delahay RJ, Lesellier S, Ashford R, Dalley D, Gowtage S, Dave D, Palmer S, Brewer J (2011). Bacillus Calmette-Guerin vaccination reduces the severity and progression of tuberculosis in badgers. PROCEEDINGS OF THE ROYAL SOCIETY B-BIOLOGICAL SCIENCES.

[92] Buddle BM, Aldwell FE, Keen DL, Parlane NA, Yates G, de Lisle GW (1997). Intraduodenal vaccination of brushtail possums with bacille Calmette-Guerin enhances immune responses and protection against Mycobacterium bovis infection. The international journal of tuberculosis and lung disease : the official journal of the International Union against Tuberculosis and Lung Disease.

[93] Gerritsen J, Smidt H, Rijkers GT, de Vos WM (2011). Intestinal microbiota in human health and disease: the impact of probiotics. Genes Nutr.

[94] Diehl GE, Longman RS, Zhang JX, Breart B, Galan C, Cuesta A, Schwab SR, Littman DR (2013). Microbiota restricts trafficking of bacteria to mesenteric lymph nodes by CX(3)CR1(hi) cells. Nature.

[95] Nave H, Gebert A, Pabst R (2001). Morphology and immunology of the human palatine tonsil. Anat Embryol (Berl).

[96] Lugton I (1999). Mucosa-associated lymphoid tissues as sites for uptake, carriage and excretion of tubercle bacilli and other pathogenic mycobacteria. Immunol Cell Biol.

[97] Fujimura Y (1986). Functional morphology of microfold cells (M cells) in Peyer's patches--phagocytosis and transport of BCG by M cells into rabbit Peyer's patches. Gastroenterol Jpn.

[98] Teitelbaum R, Schubert W, Gunther L, Kress Y, Macaluso F, Pollard JW, McMurray DN, Bloom BR (1999). The M cell as a portal of entry to the lung for the bacterial pathogen Mycobacterium tuberculosis. Immunity.

[99] Fujimura Y (2000). Evidence of M cells as portals of entry for antigens in the nasopharyngeal lymphoid tissue of humans. Virchows Arch.

[100] Brandtzaeg P (2011). Potential of nasopharynx-associated lymphoid tissue for vaccine responses in the airways. Am J Respir Crit Care Med.

[101] Kuroda K, Brown EJ, Telle WB, Russell DG, Ratliff TL (1993). Characterization of the internalization of bacillus Calmette-Guerin by human bladder tumor cells. J Clin Invest.

[102] Nochi T, Yuki Y, Matsumura A, Mejima M, Terahara K, Kim DY, Fukuyama S, Iwatsuki-Horimoto K, Kawaoka Y, Kohda T (2007). A novel M cell-specific carbohydrate-targeted mucosal vaccine effectively induces antigen-specific immune responses. J Exp Med.

