# Supplementary material for: Detection of live M. bovis BCG in tissues and IFN-γ responses in European badgers (Meles meles) vaccinated by oropharyngeal instillation or directly in the ileum
Source: BMC Vet Res. 2019 Dec 6;15:445. doi: 10.1186/s12917-019-2166-4 (PMC6898942; doi:10.1186/s12917-019-2166-4)
Supplement: Supplementary file 1 — Additional file 1: Table S1. Primers used for the detection of mycobacteria by RT-PCR [file 12917_2019_2166_MOESM1_ESM.docx]

Supplementary table: Primers used for the detection of mycobacteria by RT-PCR

| Mycobacterial species detected | Test 1  Identify M.tb complex | | Test 2  Differenciate from *M. microti* (groups C and D) | | Test 3  BCG (groups A and B) | Test 4  Mycobacterial species (groups A and B) | Test 5  *M. avium* (groups A and B) |
| --- | --- | --- | --- | --- | --- | --- | --- |
|  | IS*6110* | IS*1081* | IS*1561’* | Rv1510  (RD4) | **RD1 flanking region** | hsp65 | IS*1245* |
| *M. bovis* | √ | √ | √ | - | - | √ | - |
| *M. microti* | √ | √ | - | √ | - | √ | - |
| BCG | √ | √ | √ | - | √ | √ | √ |
| *Non tuberculous* *Mycobacterium (M. sp)* | - | - | - | - | - | √ | - |
| *M. avium* | - | - | - | - | - | √ | √ |
